# Effects of reflexology on premenstrual syndrome: a systematic review and meta-analysis

**DOI:** 10.1186/s13030-019-0165-0

**Published:** 2019-10-24

**Authors:** Marzieh Hasanpour, Mohammad Mehdi Mohammadi, Habib Shareinia

**Affiliations:** School of Nursing and Midwifery, Tehran University of Medical Sciences, Tehran, Iran

**Keywords:** Premenstrual syndrome (PMS), Reflexology, Systematic review, Massage, Meta-analysis

## Abstract

**Background:**

Premenstrual syndrome (PMS) refers to a set of somatic and psychological symptoms that occur cyclically in the luteal phase of a menstrual cycle. There is no report of final result of reflexology on PMS. Therefore, the present study aimed to determine the effect of reflexology on PMS through a systematic review and meta-analysis study.

**Method:**

The present study was a systematic review and meta-analysis that was conducted by searching in 8 electronic databases including PubMed, EMBASE, Cochrane Library, Web of Science, ProQuest, Scopus, Google Scholar, and SID until December 28, 2018. In this regard, interventional studies, which examined the impact of reflexology on women with premenstrual syndrome, were included. These studies were published during 1993 to 2018. The Cochrane Collaboration’s Risk of Bias Tool was used to assess the quality of studies. Meta-analysis was performed by the help of CMA 2 software.

**Results:**

Nine out of 407 studies finally remained after screening, and quantitative and quantitative analyses were performed on them. The total number of research samples was 475. The mean treatment time with reflexology was 40.55 min per session that was performed in 6 to 10 sessions of treatment in 66.67% of studies. According to the meta-analysis and based on the random effects model, the reflexology could decrease the severity of PMS in the intervention group compared to the control group (SMD = − 2.717, 95% CI: − 3.722 to − 1.712). Meta-regression results indicated that the duration of intervention sessions (β = − 0.1124, 95% CI − 0.142 to − 0.084, *p* < 0.001) had a significant impact on the severity of PMS. Reflexology could also significantly affect somatic (SMD = − 1.142, 95% CI: − 1.481 to − 0.803) and psychological (SMD = − 1.380, 95% CI: − 2.082 to − 0.677) symptoms arising from PMS.

**Conclusion:**

In general, results of the present study indicated that the reflexology could relieve PMS symptoms, so that overall scores, somatic and psychological symptoms of PMS decreased by applying the reflexology intervention. Furthermore, an increase in the length of reflexology time in each session increased its efficiency. Reflexology can be used as an effective intervention in a patient care program by nurses and its efficiency can be enhanced by increasing intervention time in each reflexology treatment session.

## Background

Premenstrual syndrome (PMS) refers to a set of somatic and psychological symptoms that occur cyclically in the luteal phase of a menstrual cycle [1]. PMS was first introduced as a diagnostic concept by Raymond Greene and Katharina Dalton in 1953 [2]. The global prevalence of this syndrome is 47.8% in the world. Some countries have reported the prevalence of PMS as follows: Spain: 73%, Switzerland: 19%, China: 21%, Brazil: 60%, and India: 67% [3]. According to the evidence, this prevalence is 98% in Iran [4].

Clinical symptoms of PMS can be divided into two categories: somatic and psychological. The symptoms occur cyclically just before menstruation and disappear when menstrual bleeding starts. Major somatic symptoms include swelling, breast tenderness, headache, increased appetite and palpitations; and psychological symptoms include depression, irritability, fatigue, aggression, suicidal tendency, and social isolation [5].

PMS can affect women’s quality of life, so that most of these women have somatic problems (dizziness, headache, nausea, palpitation, sweating, pain, weakness, and lethargy) and psychological problems (anxiety, anger, depression, irritability, isolation, stress, and impatience) [6].

The exact cause of this syndrome is unknown, and symptoms are reported such as changes at estrogen and progesterone levels, central changes in catecholamines, response to prostaglandins, reduction of dopamine and central serotonin levels what are now more taken into consideration. There is no effective single treatment, which is universally accepted, for this syndrome [7].

PMS can be treated by pharmacological and non-pharmacological treatments. The pharmacological treatment include diuretics, gonadotropin-releasing hormone (GnRH) agonist, and non-steroidal anti-inflammatory drugs (NSAIDs) and the main pharmacological treatments include combined oral contraceptives (COCs) and selective serotonin reuptake inhibitors (SSRIs) [8]. The prescribed drugs for this purpose are associated with undesirable effects such as fatigue, headache, irritability, depression and gastrointestinal bleeding [9, 10]. Non-pharmacological methods, which are called complementary therapies, such as the reflexology are more secure and have fewer complications than pharmaceutical methods [11, 12].

Reflexology is a systematic function based on which points of hands, legs, or ears are placed under some pressure. It is based on the fact that the stimulation of reflex points on palms, legs, and ears matches each part of the body including muscle, nerve, gland and bone. In other words, reflex points indicate functions of various organs of the body. When reflex points are stimulated, body cells affect the health of a body organ, which is associated with that reflex point, by creating a reflex impact [13]. The reflexology therapy mechanism can be studied based on its main origin, so that Traditional Chinese Medicine (TCM) expresses that under this mechanism, the stimulation of reflex points leads to the restoration, reconstruction, and balance of vital Qi energy, thereby treating diseases [14].. In general, the precise mechanism of reflexology operation is still unknown, but there are different theories on the efficiency of reflexology. According to the regional theory, there are certain reflexes in hands, ears, and legs and they are associated with glands, organs, and parts of the body by energy channels or meridians. It is believed that these energy channels are blocked during illness or imbalance in the body. Opening these blocked paths, reflexology massage leads to a free flow of energy in the body and thus the body regain its health and balance. Based on the theory of neural message, the reflexology inhibits the transmission of pain by controlling the transmission of afferent nerve signals and closure of nerve valve in the posterior branch of the spinal cord [14, 15].

There is no final result of reflexology’s impact on PMS. In other words, the scientific community needs to create a clear insight into the effect of reflexology on PMS. A systematic review and meta-analysis study can summarize the results of previous studies and provide a clear result in this regard. Given that there was no systematic review and meta-analysis study on this field, the present study aimed to determine the impact of reflexology on PMS in women.

## Methods

### Search strategy

The present study was conducted in accordance with the Preferred Reporting Items for Systematic Reviews and Meta-analyses (PRISMA) statement [16]. In this regard, 8 electronic databases including PubMed, EMBASE, Cochrane Library, Web of Science, ProQuest, Scopus, Google Scholar, and SID were searched until December 28, 2018. The applied keywords in the search included Premenstrual Syndrome; Premenstrual Complaints; Reflexology; Zone Therapy; Massage; Menstruation; Menstrual, and Premenstrual Symptoms. In addition, references of all studies were manually searched and reviewed in order to ensure a comprehensive search. All references were made by two authors, who independently searched, and the results were then merged. The full search strategy was attached to the research (Additional file 1).

### Inclusion and exclusion criteria

Studies, which had the following criteria, were eligible:

1. Type of design: RCT (Randomized Controlled Trial) or Quasi-experimental designs
2. Population: Participants with PMS
3. Intervetion group: Reflexology as intervention
4. Outcome: Overall scores, somatic or psychological symptoms of PMS are measured and reported.
5. Control group: Placebo or non-treatment are considered in this group.

Studies, which used reflexology in combination with other interventions (e.g. relaxation), were excluded from the research. Furthermore, studies without a control or comparison group were excluded.

### Study selection method

A strategy, which was proportional to each database, was used to search studies. The manual search was also performed to find thesis, dissertation and conference proceedings. Duplicated studies were deleted by EndNote software, and what remained was under the initial screening. In this regard, the eligibility of studies was independently examined by two authors; and any disagreement between two investigators was resolved by the third party. At the first stage, titles and abstracts of studies were first assesed for the initial screening; and each author independently selected studies with the inclusion criteria, and then remaining studies were selected through a full-text study. Finally, a quantitative and qualitative analysis was performed on remaining studies.

### Quality of studies

The Cochrane Collaboration’s Risk of Bias Tool was utilized to assess the quality of studies [17]. The risk of bias was rated using a Low/High/Unclear Grading Scale in studies (Table 1). It should be noted that two authors independently studied the quality of studies.

**Table 1:** Risk of Bias of Included Studies*

^(Citation)^ Study (Year)	Selection Bias		Patient Blinding	Assessor Blinding	Incomplete Outcome Data	Selective Outcome Reporting
	Random Sequence Generation	Allocation Concealment				
[18] Oleson (1993)	L	U	L	U	L	L
[19] Kim (2002)	H	H	U	U	L	L
[20] Kim (2004)	U	U	U	U	U	L
[21] Lee (2011)	H	H	U	U	L	L
[11] Abdollahi Fard (2013)	L	U	L	U	L	L
[22] Baghdassarians (2015)	U	U	U	U	U	U
[23] Nalini (2015)	U	U	U	U	U	L
[24] Prema (2017)	U	U	U	U	U	U
[12] Shafaie (2018)	L	U	L	H	L	L

*Domains of Quality Assessment Based on the Cochrane Tools for Assessing Risk of Bias

Abbreviations; *L* low Risk of Bias, *H* High Risk of Bias, *U* Unclear (Uncertain) Risk of Bias

One study used a random number table for the Random Sequence Generation [18]. Two studies used software for random allocation [11, 12]. Four studies did not clearly report the Random Sequence Generation [20, 22–24] and two other studies considered High Risk of Bias [19, 21].

Seven studies did not clearly report the allocation concealment [11, 12, 18, 20, 22–24], and two studies considered High Risk of Bias [19, 21].

Blinding was evaluated separately for outcome assessors and patients. In three studies, patients did not know which of intervention or control groups they were allocated [11, 12, 18]. There was no possibility of judgment in other studies due to poor reporting [19–24]. About blinding outcome assessors, one research directly reported that blinding was only performed on samples and data analyzers; and outcome assessors were not blind [12]. Outcome assessor blinding was not clearly reported in other studies [11, 18–24]. Table 1 presents other examined dimensions.

### Data extraction method

Two authors independently extracted data. In this regard, a data extraction form was used including the author’s name (year of publication), country, design study, sample size, type of intervention, number of sessions, and duration of each session, research group, age range (or mean age) of measured outcomes and measurement tools.

### Statistical analysis method

In the present study, the meta-analysis was done using Comprehensive Meta-Analysis software (CMA, Version 2.0, New England, NJ, USA). The standardized difference was calculated with 95% confidence interval as the effect size. In the study, a random effects model was used; and the heterogeneity of studies was analyzed using I^2^ value. The analysis sub-group and meta-regression were used to further investigate the heterogeneity source; and included studies were allocated to sub-groups in terms of country, studied age group, study design, type of comparison group, and type of reflexology. In order to perform the meta-regression, duration of intervention sessions and the study publication year were considered as moderating variables. In order to calculate the Pre-Post Correlation, the Standard Deviation of Changes (SD_change_) was extracted based on a study by Lee et al. [21], and then, the Pre-Post Correlation was estimated at 0.803 using the following formula, and finally generalized to other studies.

r = (SD_pre_ ^ 2 + SD_post_ ^ 2 - SD_change_ ^ 2) / (2 * SD_pre_ * SD_post_)

Publication Bias was examined using the Funnel Plot, and a test by Begg and Egger; and the trim and fill method was then used to measure the Adjusted Effect Size if there was a publication bias. Sensitivity analysis was used to investigate the robustness of results.

## Results

### Study selection

**Fig. 1:**
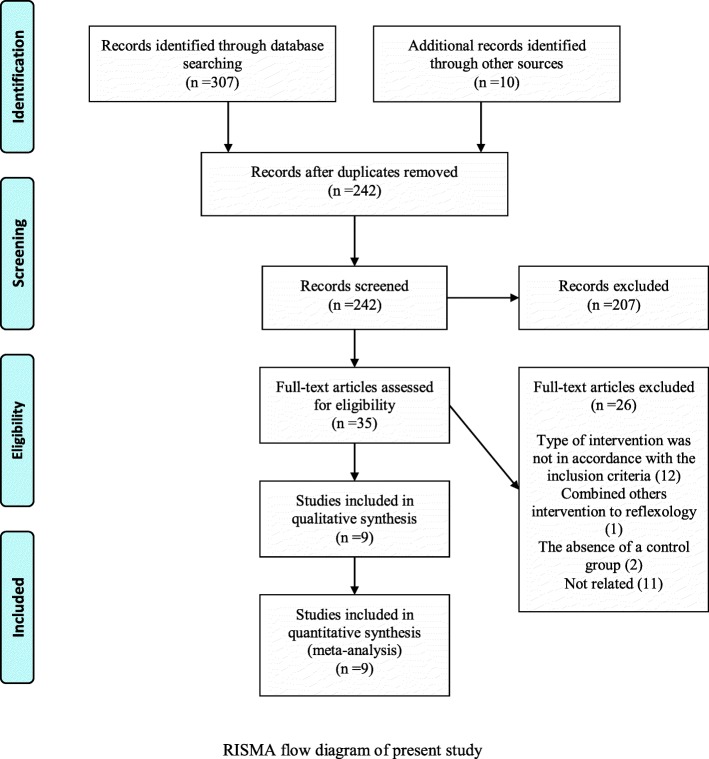
RISMA flow diagram of present study

The applied search strategy in databases detected 307 studies; and 10 other studies were then added to them after manual search of references and complementary search in theses and conferences. After removal of duplicated studies (*n* = 75), the research screening began with 242 studies; hence, 207 studies were excluded from the study by evaluating their titles and abstracts. The full text of 35 papers was then studied, and 26 studies were excluded due to the lack of compliance of intervention type with the inclusion criteria and the lack of control group. Finally, 9 remained studies were investigated by qualitative and quantitative analyses (Fig. 1).

### Study characteristics

Nine studies (6 RCT and 3 Quasi- experimental studies published during 1993 to 2018) were selected for the present study. The total number of study samples was 475. Studies were conducted in the United States [18], North Korea [19–21], India [23, 24], and Iran [11, 12, 22]. A total of 3 studies were published in English [18, 23, 24, and], 3 in Persian [11, 12, 22] and 3 in Korean [19–21]. Six studies were randomized clinical trials [11, 12, 18, 20, 22, 24] and three ones were semi experimental [19, 21, 23]. The average treatment time with reflexology was 40.55 min per session that was performed in 6 to 10 treatment sessions in 66.67% of studies. Table 2 presents other details of included studies.

**Table 2:** Characteristics of included studies

Author (Publication year)	Country	Design of Study	Sample Size /Group	Type of Intervention	Intervention Time Schedule	Population (Mean or age range)	Outcomes Measures	Instrument
Oleson (1993)	USA	RCT	35 EG:18 CG:17	EG: Foot, hand and ear reflexology; CG: Placebo reflexology	30 min	Females with Premenstrual symptoms (Mean age: EG:37.2; CG:32.7)	Total PMS; Somatic symbtoms; Psychological symbtoms	PMS scale (made by researcher)
Kim (2002)	South Korea	Quasi expermental desin	40 EG:20 CG:20	EG: Foot reflexology; CG: no treatment	60 min	Female college student (range: 21–33 years)	Total PMS and dysmenorrhea	Keele VAS and opening records
Kim (2004)	South Korea	RCT	48 EG:24 CG:24	EG: Self foot reflexology; CG: no treatment	35 min	High school girls (range: U)	Total PMS; Behavioral symbtoms; Psychological symbtoms; Dysmenorrhea	MDQ; VAS
Lee (2011)	South Korea	Quasi expermental desin	61 EG:37 CG:24	EG: Aroma-foot-reflexology; CG: no treatment	60 min	Female college student (Mean age: EG:19.3; CG19.4)	Total PMS and dysmenorrhea; Lower abdominalskin temperature	PMS scale (made by researcher); VAS; Portable digital skin thermometer
Abdollahi Fard (2013)	Iran	Single blind RCT	90 EG:45 CG:45	EG: Foot reflexology; CG: Placebo reflexology	30 min	Female college student (Mean age: EG:20.8; CG:20.5)	Total PMS; Somatic symbtoms; Psychological symbtoms	Daily record scale
Baghdassari (2015)	Iran	RCT	40 EG:20 CG:20	EG: Foot reflexology; CG: no treatment	60 min	Females with Premenstrual symptoms (Range: 31–45)	Total PMS	PMS scale
Nalini (2015)	India	Quasi experimental	30 EG:15 CG:15	EG: Foot, hand reflexology; CG: no treatment	40 min	Female college student (Range: 17–20)	Total PMS; Somatic symbtoms; Psychological symbtoms; Emotional symptoms; Physiological symptoms	PMS scale
Prema (2017)	India	RCT	30 EG:15 CG:15	EG: Foot reflexology; CG: no treatment	20 min	Adolescent girls (Mean age: EG:U; CG:U)	Total PMS; Psychological symbtoms	VAS
Shafaie (2018)	Iran	Double blind RCT	101 EG:52 CG:49	EG: Foot reflexology; CG: Placebo reflexology	30 min	Female college student (Mean age: EG:22.3; CG:21.46)	Total PMS; Somatic symbtoms; Behavioral symptoms: Psychological symbtoms	Daily record scale

EG: Experimental group; CG: Control group; PMS: Premenstrual syndrome; RCT: Randomized controlled trial; MDQ: Menstural Distress Questionnaire; VAS: Visual analogue scale; U: unclear

### Effect of reflexology on overall score of PMS

**Fig. 2:**
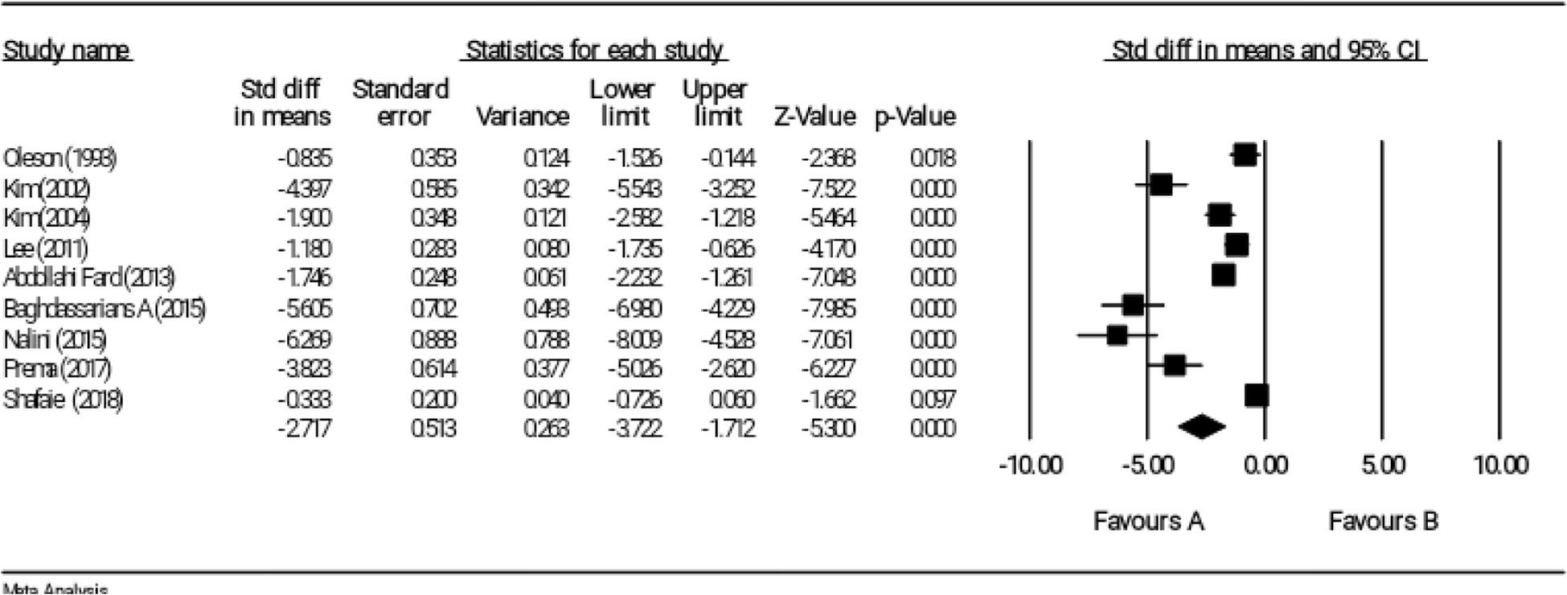
Effect of reflexology on overall score of PMS

All nine selected studies examined the impact of reflexology on overall score of PMS [11, 12, 18–24]. According to the meta-analysis and based on random effects model, the reflexology could be decreasing the severity of PMS in the intervention group compared to the control group (SMD = − 2.717, 95% CI: − 3.722 to − 1.712); however, Shafaie et al. (SMD = − 0.333, 95% CI: − 0.726 to 0.060) did not reported any significant result (Fig. 2). Heterogeneity of studies was also significant (I^2^ = 94.41%, *P* < 0.001).

### Examination of heterogeneity source

**Fig. 3:**
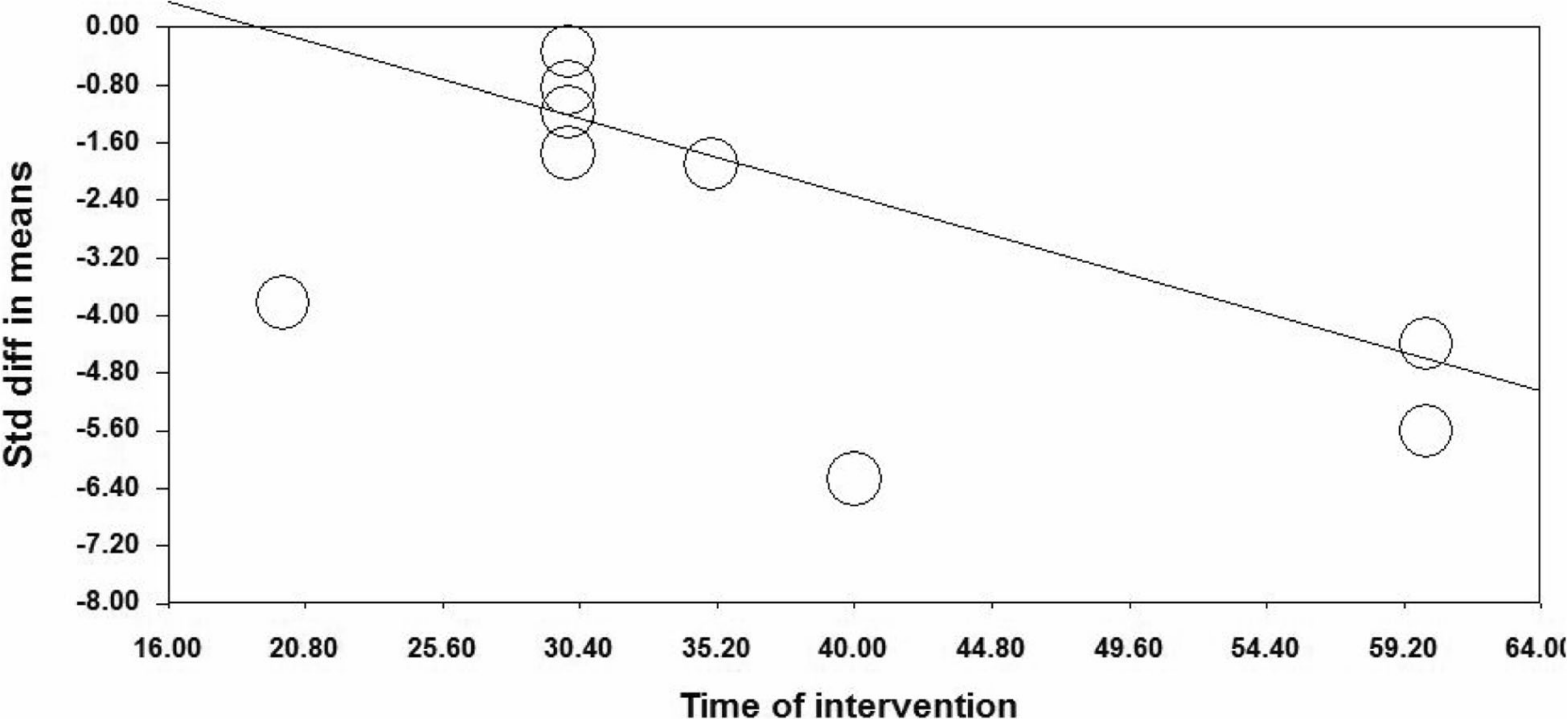
Meta-regression of intervention duration on SMD in the studies investigating the effect of reflexology on total score of PMS

The subgroup analysis and meta-regression were used to investigate source of heterogeneity. Subgroup analysis was performed based on research year, country, type of research design, type of comparison group, type of reflexology, and age group of research participants; however, results of the subgroup analysis indicated that the source of heterogeneity was not resulted from the above factors. In order to perform the meta-regression, duration of intervention sessions and the research publication years were considered as moderating variables. Meta-regression results indicated that duration of intervention sessions (β = − 0.1124, 95% CI − 0.142 to − 0.084, *p* < 0.001) had a significant impact on severity of PMS (Fig. 3); however, publication year did not report any significant impact (β = 0.022, 95% CI − 0.007 to − 0.051, *p* = 0.129).

### Publication Bias

**Fig. 4:**
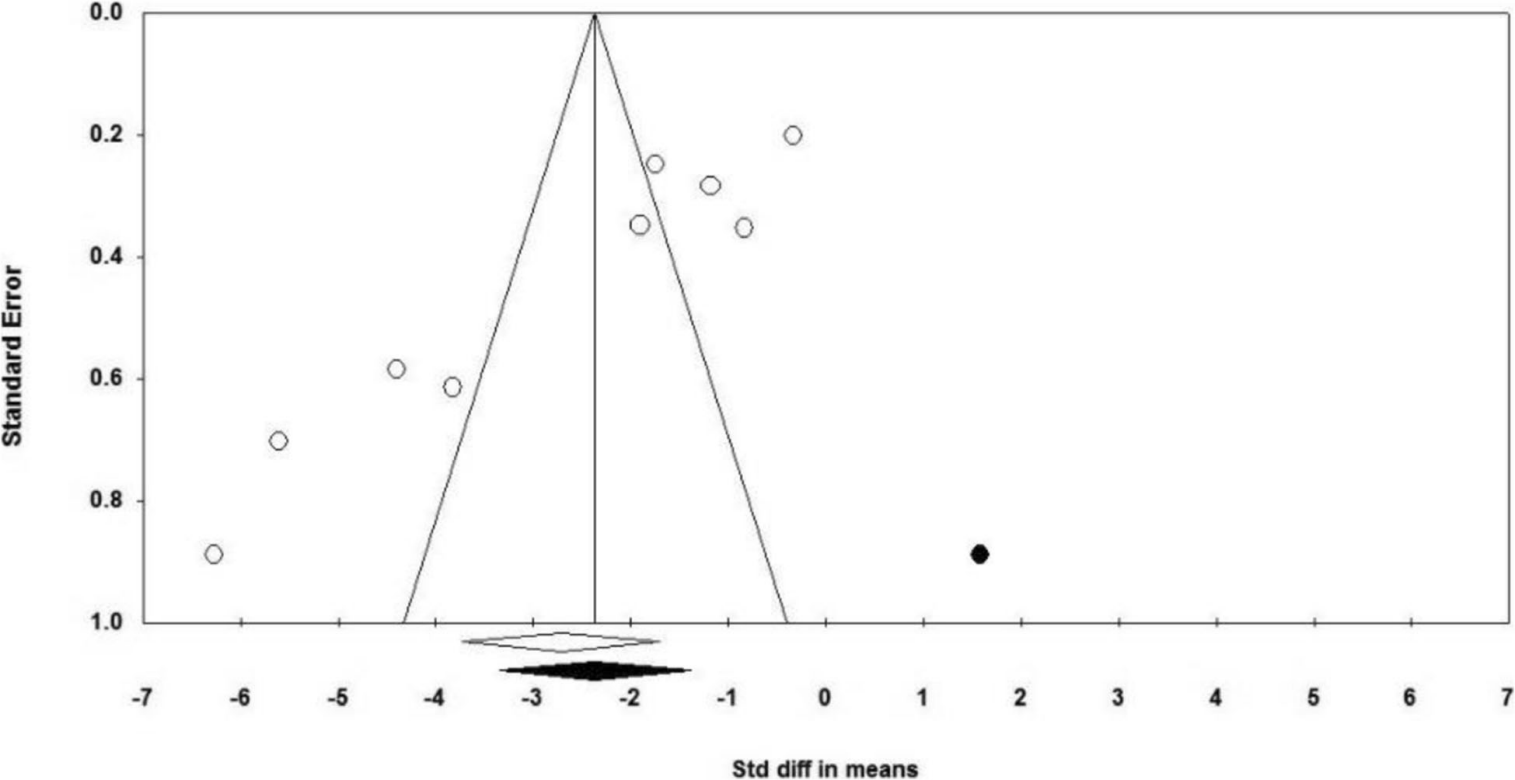
Funnel plot for publication bias in the studies investigating the effect of reflexology on total score of PMS

The Funnel Plot visually showed the probability of publication bias (Fig. 4), and then the bias was examined based on tests by Begg and Egger; and the publication bias was reported in both tests (Begg’s test, *P* = 0.013; Egger’s test, *P* = 0.001).

Therefore, the trim and fill method was used; accordingly, 1 study was allocated and the effect size was − 2.365 that was changed by 0.335 compared to the observed effect size (− 2.718), and in generally had no effect on significance of effect size.

### Sensitivity analysis

**Fig. 5:**
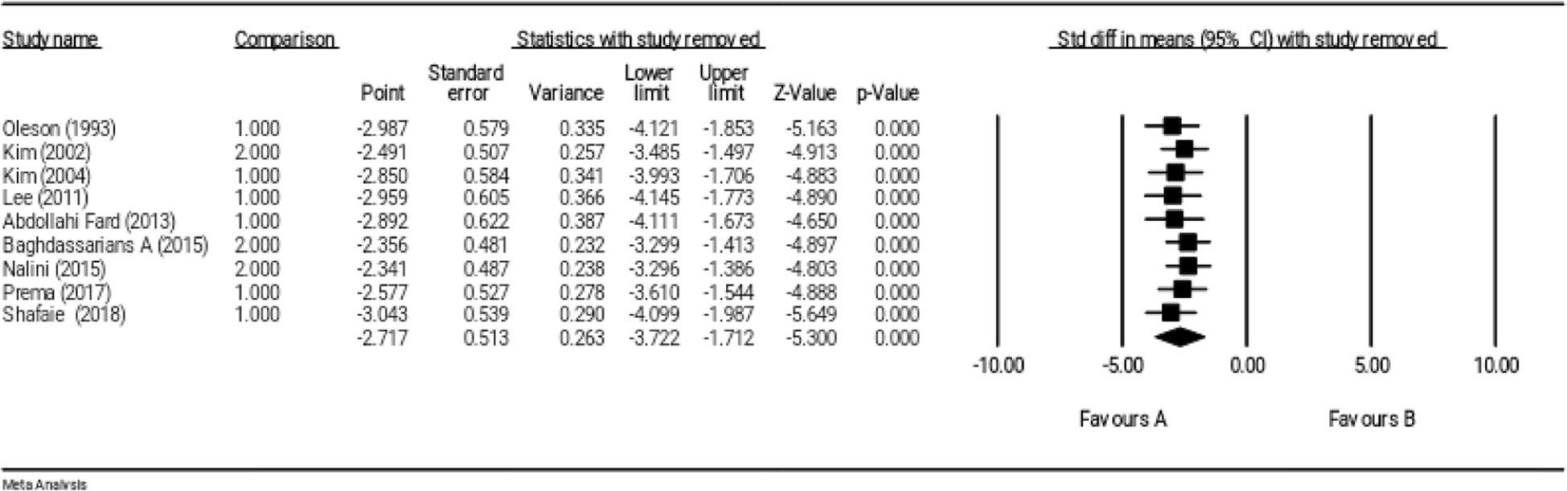
Sensitivity analysis for studies investigating the effect of reflexology on total score of PMS

The robustness of the primary results from 9 studies was supported by the sensitivity analysis; and an estimation of robustness of overall effect size was obtained by removing any study from the meta-analysis. In other words, the sensitivity analysis indicated that the exclusion of results of each study from the general analysis did not have any significant effect on overall results (Fig. 5).

### Effect of reflexology on somatic symptoms of PMS

Four studies investigated somatic symptoms of PMS as outcomes of study [11, 12, 18, 23]. These studies included a total of 256 samples. The age range of participants was mostly from 17 to 38 years. The duration of each session of reflexology treatment was 32.5 min on average in papers.

**Fig. 6:**
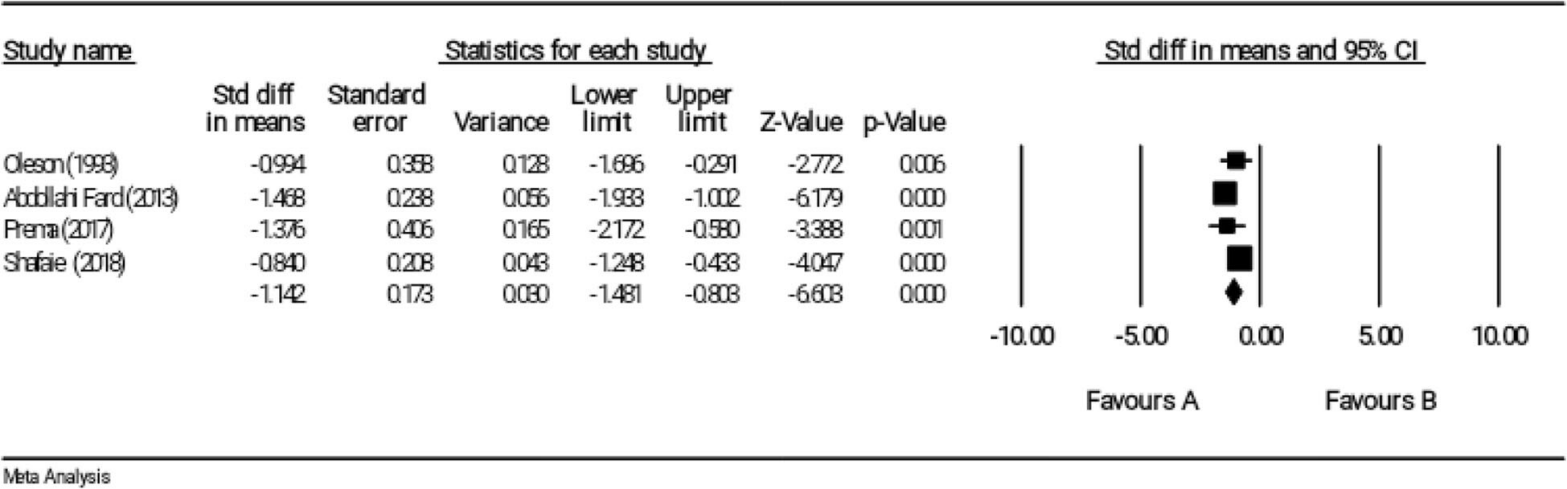
Effect of reflexology on somatic symptoms of PMS

Results of the meta-analysis indicated that the reflexology could significantly affect somatic symptoms of PMS based on the random effects model. (SMD = − 1.142, 95% CI: − 1.481 to − 0.803); (Fig. 6). Furthermore, no significant heterogeneity was reported (I^2^ = 32.94%, *P* = 0215).

### Publication Bias

**Fig. 7:**
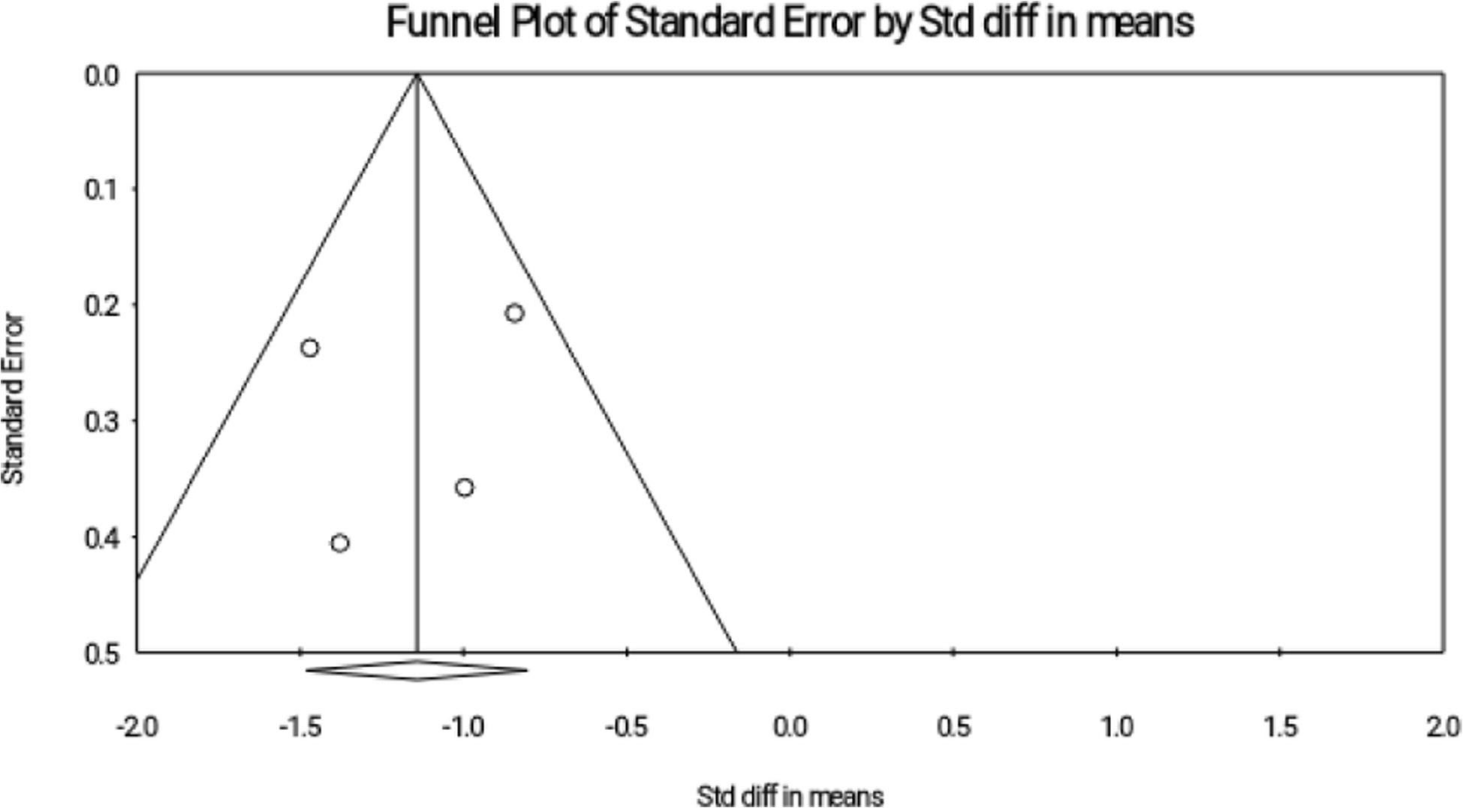
Funnel plot for publication bias in the studies investigating the effect of reflexology on somatic symptoms of PMS

The funnel plot visually showed that there was no publication bias (Fig. 7). However, Egger and Begg tests also indicated that there was no publication bias (Begg’s test, *P* = 0.497; Egger’s test, *P* = 0.704).

### Sensitivity analysis

**Fig. 8:**
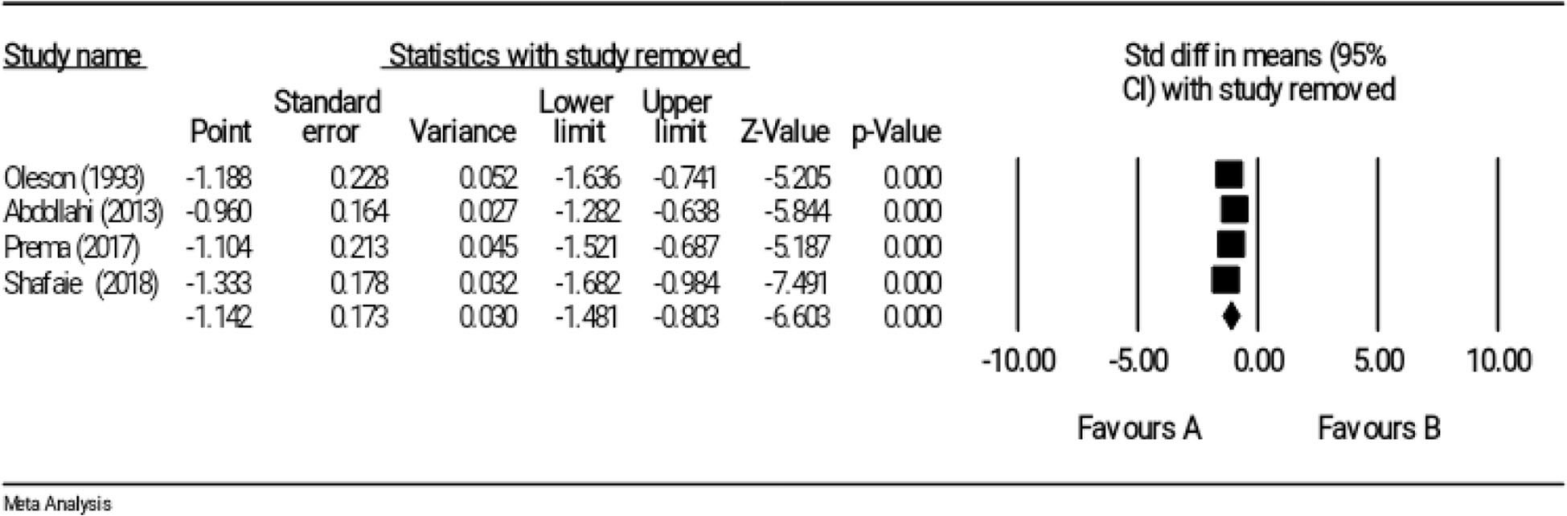
Sensitivity analysis for studies investigating the effect of reflexology on somatic symptoms of PMS

The sensitivity analysis indicated that the exclusion of any included study in the meta-analysis would not change overall effect size (Fig. 8).

### Effect of reflexology on psychological symptoms of PMS

Six studies investigated psychological symptoms of PMS as an outcome [11, 12, 18, 20, 23, 24]; so that, they measured psychological symptoms of PMS in addition to examining the impact of reflexology on overall score of PMS. The studies included a total of 334 participants in the age group of 17 to 38 years. The mean intervention time was 30.84 per session in the studies.

**Fig. 9:**
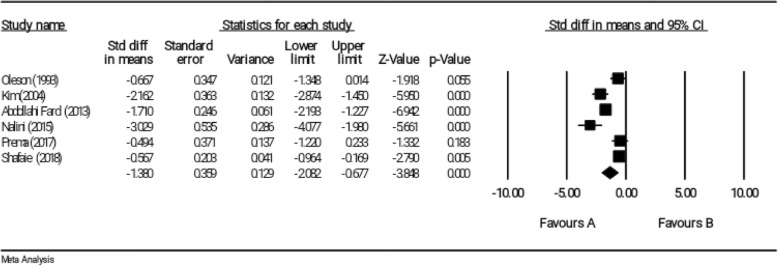
Effect of reflexology on psychological symptoms of PMS

According to meta-analysis result and based on the random effects model, the reflexology could significantly decrease psychological symptoms of PMS in the intervention group compared with the control group (SMD = − 1.380, 95% CI: − 2.082 to − 0.677); however, a study (SMD = − 0.494, 95% CI: − 1.220 to 0.233) by Prema et al. did not report any significant result (Fig. 9). The results indicated that the heterogeneity was significant (I^2^ = 87.05%, *P* < 0.001).

### Heterogeneity source

**Fig. 10:**
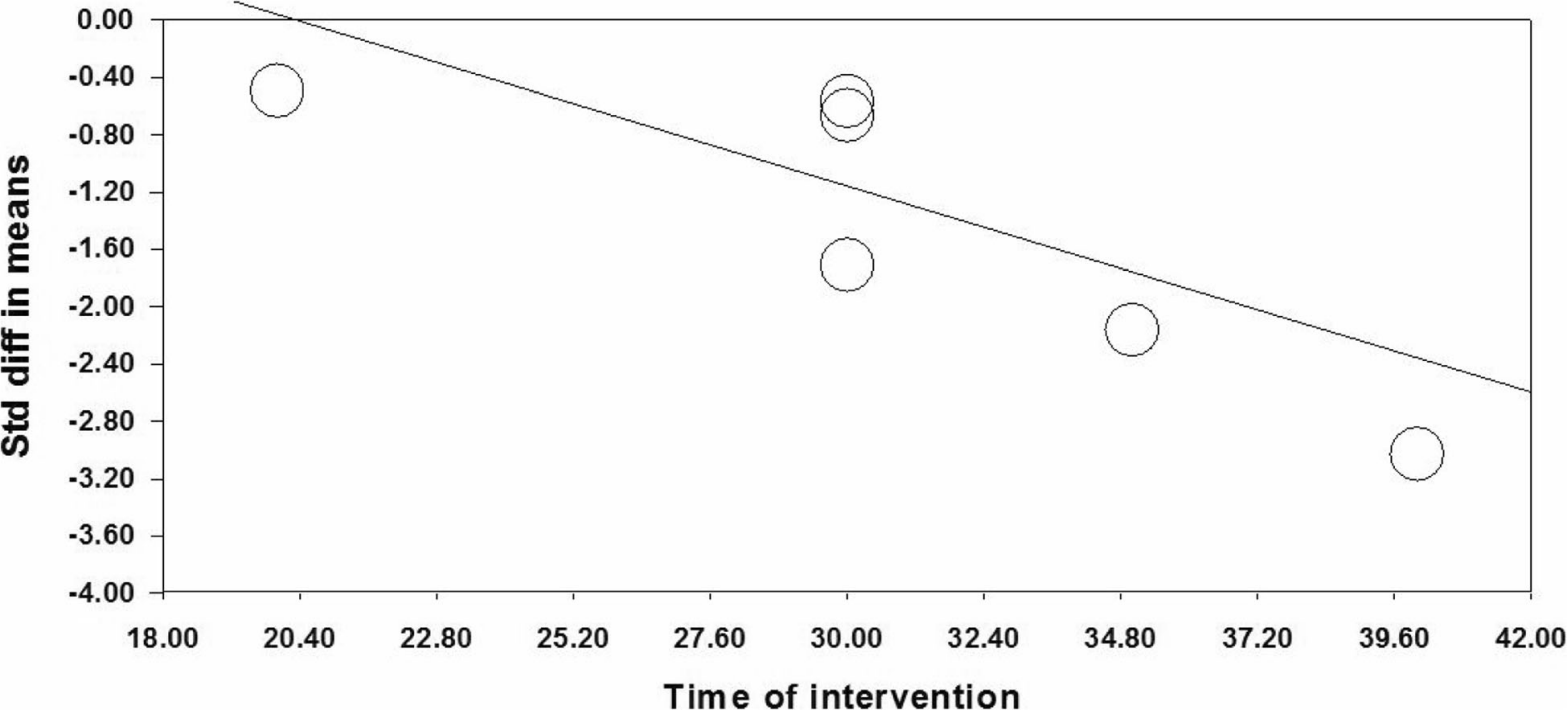
Meta-regression of intervention duration on SMD in the studies investigating the effect of reflexology on psychological symptoms of PMS

It seems that duration of intervention is the source of heterogeneity in different studies as the meta-regression results indicate a significant effect of duration of intervention on psychological symptoms of PMS (β = − 0.119, 95% CI − 0.175 to − 0.065, *p* < 0.001) (Fig. 10).

### Publication Bias

**Fig. 11:**
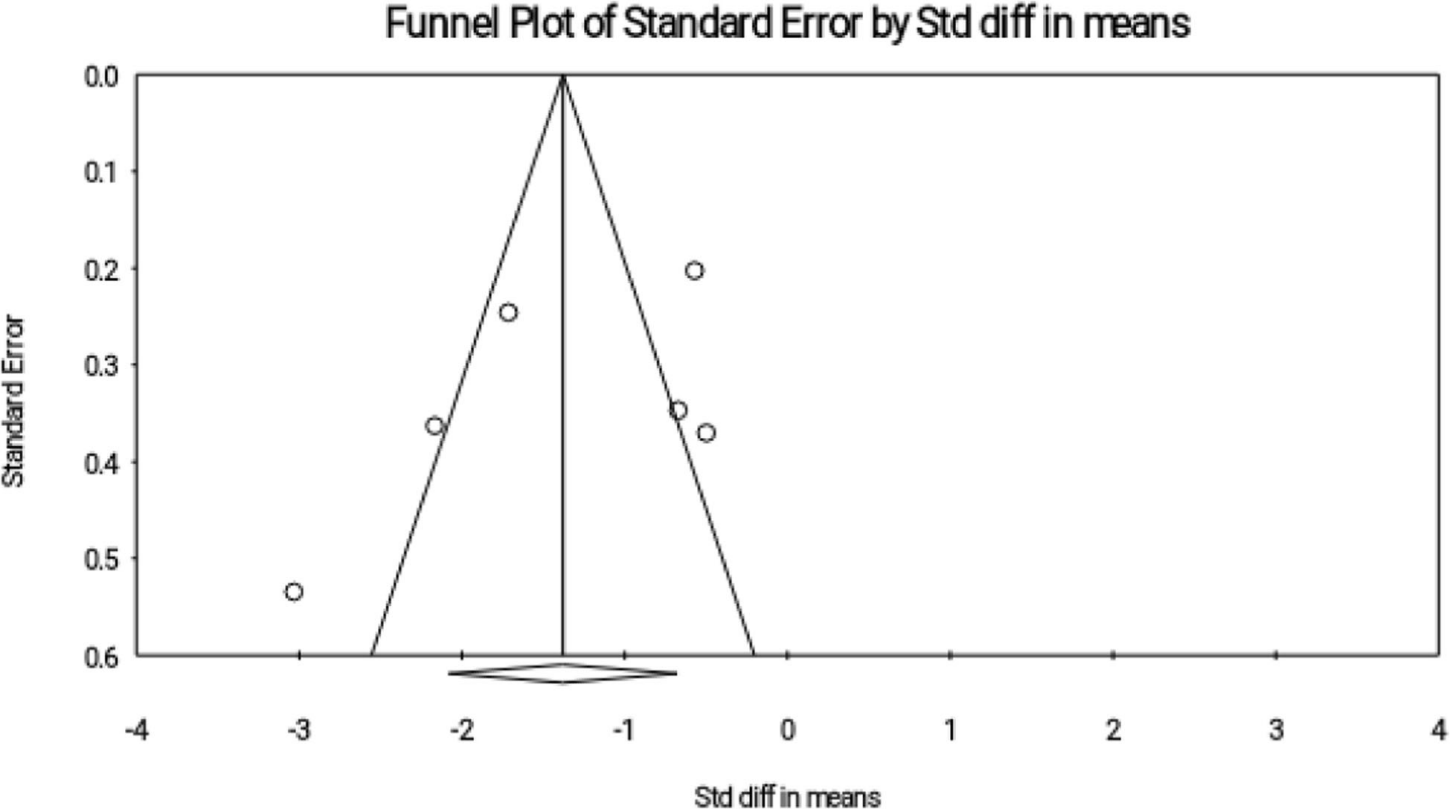
Funnel plot for publication bias in the studies investigating the effect of reflexology on psychological symptoms of PMS

Funnel plot and Begg and Egger test did not report any publication bias (Fig. 11). (Begg’s test, *P* = 0.189; Egger’s test, *P* = 0.287).

### Sensitivity test

**Fig. 12:**
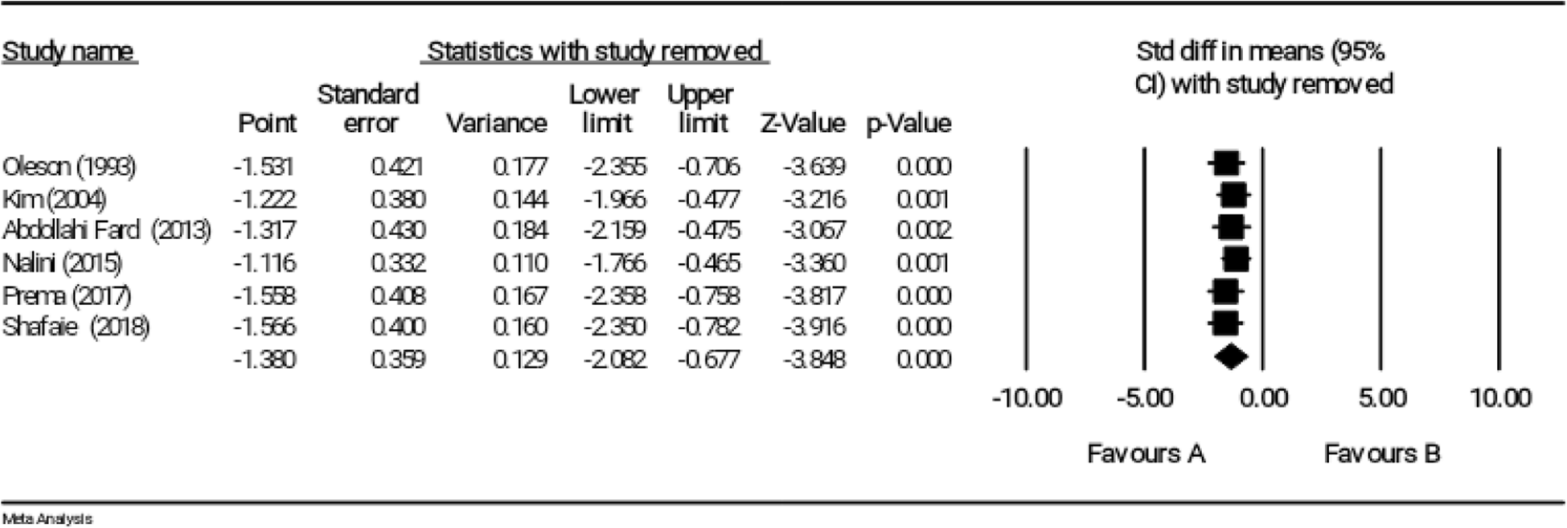
Sensitivity analysis for studies investigating the effect of reflexology on psychological symptoms of PMS

Results of the sensitivity test indicated that the exclusion of each study did not affect the significance of overall effect size (Fig. 12).

## Discussion

### Summary of evidence

The present systematic review and meta-analysis study indicated that the reflexology as an intervention could decrease the severity of PMS symptoms in women. In this regard, we analyzed 9 studies that measured the impact of reflexology on the overall score of PMS. Meta-analysis results for overall score of PMS indicated that the reflexology could effectively relieve overall symptoms of PMS; however, effect sizes of various studies were heterogeneous. The duration of reflexology in each intervention session could explain the heterogeneity; in other words, the increased duration of using a reflexology intervention in each session enhanced its effect on the overall score of PMS. Lee and Baghdasar introduced 60 min as the standard time for reflexology with the greatest effect on overall score of PMS [21, 22]. It should be noted that none of 9 reported studies reported any side effect for the reflexology intervention.

The present study also indicated that the reflexology could decrease the severity of somatic symptoms of PMS. In this regard, a meta-analysis was performed on four studies with homogeneous effect sizes. There was no systematic review and meta-analysis study on the effect of reflexology on somatic symptoms of PMS; however, the effect of reflexology was investigated on some somatic symptoms in other demographic groups. In this regard, Lee et al. (2011) examined the impact of reflexology on pain, fatigue and sleep in various groups of nurses, students, elderly patients and patients. Their results indicated that the reflexology as an effective intervention could relieve fatigue and improve sleep quality, but its effect on pain was not significant in some population groups [25]. Despite the fact that the reflexology dates back to Traditional Chinese Medicine (TCM), conventional sciences have also presented its effectiveness mechanism, so that reflexology induces a sense of meditative relaxation that stimulates the parasympathetic system; hence, it seems that its impact on somatic symptoms of PMS is due to the improved parasympathetic functions of some body systems [14].

Other results of the present study indicated that the reflexology led to the improvement of psychological symptoms of PMS. However, effect sizes of various studies were heterogeneous. Further examination indicated that duration of each session of reflexology was the cause of this heterogeneity terms of psychological symptoms. In this regard, the increased intervention time of each session enhanced the impact of reflexology on the relief of psychological symptoms. There was no systematic review and meta-analysis study on the impact of reflexology on psychological symptoms of PMS; however, Chandrababu et al. (2019) conducted a systematic and review and meta-analysis study and found that the reflexology could reduce the anxiety of patients undergoing cardiovascular interventions [13]. The proposed mechanism of contractual medicine regarding the effectiveness of reflexology on psychological symptoms can be due to the fact that reflexology leads to the release of β-endorphins and encephalins from the brain as endogenous opiate peptides with Euphoriant effects that promote a good sense in people [14].

The robustness of results was supported for all three outcomes, namely total score, somatic and psychological symptoms of PMS; and an estimation of robustness overall effect size was obtained by removing any study from the meta-analysis. In other words, each study alone was not able to change overall results, and the significance of overall result was not affected by the outcome of a study.

Results of the present study provided valuable evidence for nurses as health care providers. Nurses can use the research results to improve symptoms of PMS in women. On the other hand, none of studies provided any adverse effects of reflexology, thereby indicating the importance of using the reflexology as an effective and less-complicated treatment. On the other hand, health policymakers can use the reflexology as a complementary and alternative therapy in the caregiver program.

### Limitations

A limitation of study was the low number of studies that directly measured and reported somatic symptoms as outcomes; Therefore, the performance of applied statistical tests was somewhat affected to examine the publication bias in the studies. Another limitation was the publication bias in studies that examined the impact of reflexology on overall score of PMS. In this regard, adjusted effect size was measured by applying the trim and fill method. Another limitation of study was the poor and incomplete report of studies in study design and participants’ age. In some studies, we received more detailed information by emailing authors.

## Conclusion

In general, results of the present study indicated that the reflexology could relieve symptoms of PMS, so that overall score, somatic and psychological symptoms of PMS decreased by application of a reflexology intervention. Furthermore, the increased duration of reflexology in each session increased its efficiency. In this regard, it is suggested increasing the efficiency of reflexology by increasing intervention time of each session. The reflexology, as an effective intervention, can be utilized by nurses in the caregiver program. A logical step towards the future clinical trials is to compare reflexology interventions with other complementary and alternative therapies in order to provide a systematic review and meta-analysis study in this regard.

## Additional file

**Additional file 1.** The search strategy used in the systematic review. (DOCX 14 kb)

## Data Availability

The datasets used during the current study are available from the corresponding author on reasonable request.
