# Supplementary material for: Effects of reflexology on premenstrual syndrome: a systematic review and meta-analysis
Source: Biopsychosoc Med. 2019 Oct 24;13:25. doi: 10.1186/s13030-019-0165-0 (PMC6815051; doi:10.1186/s13030-019-0165-0)
Supplement: Supplementary file 1 — Additional file 1. The search strategy used in the systematic review. (DOCX 14 kb) [file 13030_2019_165_MOESM1_ESM.docx]

**Pubmed**

(((((reflexology massage[Title/Abstract]) OR reflexology[Title/Abstract]) OR zone therapy[Title/Abstract]) OR massage[MeSH Terms])) AND ((((((((premenstrual syndrome[Title/Abstract]) OR premenstrual symptoms[Title/Abstract]) OR premenstrual complaints[Title/Abstract]) OR premenstrual[Title/Abstract]) OR pre-menstrual[Title/Abstract]) OR menstruation[Title/Abstract]) OR menstrual[Title/Abstract]) OR Premenstrual Syndrome[MeSH Terms])

**27 Paper**

**2018-12-27**

**Scopus**

(TITLE-ABS-KEY(reflexology massage) OR TITLE-ABS-KEY(reflexology)  OR TITLE-ABS-KEY(zone therapy) OR TITLE-ABS-KEY(massage)) AND (TITLE-ABS-KEY(premenstrual syndrome) OR TITLE-ABS-KEY(premenstrual symptoms) OR TITLE-ABS-KEY(premenstrual complaints) OR TITLE-ABS-KEY(premenstrual) OR TITLE-ABS-KEY(pre-menstrual) OR TITLE-ABS-KEY(menstruation) OR TITLE-ABS-KEY(menstrual))

**207 Paper**

**ISI**

(TS=(reflexology massage) OR TS=(reflexology) OR TS=(zone therapy)) AND (TS=( premenstrual syndrome) OR TS=(premenstrual symptoms) OR TS=(premenstrual complaints) OR TS=(premenstrual) OR TS=(pre-menstrual) OR TS=(menstruation) OR TS=(menstrual))

**33 Paper**

**Proquest:**

(ti(reflexology massage) OR ti(reflexology) OR ti(zone therapy) OR ab(reflexology massage) OR ab(reflexology) OR ab(zone therapy)) AND (ti(premenstrual syndrome) OR ti(premenstrual symptoms) OR ti(premenstrual) OR ti(pre-menstrual) OR ti(menstruation) OR ti(menstrual) OR ti(premenstrual complaints) OR ab(premenstrual syndrome) OR ab(premenstrual symptoms) OR ab(premenstrual) OR ab(pre-menstrual) OR ab(menstruation) OR ab(menstrual) OR ab(premenstrual complaints))

**37 Paper**

**Embase**

('reflexology massage':ab,ti OR 'reflexology'/exp/mj OR 'zone therapy':ab,ti) AND ('premenstrual syndrome'/exp/mj OR 'premenstrual':ab,ti OR 'pre-menstrual':ab,ti OR 'menstruation':ab,ti OR 'premenstrual complaints':ab,ti OR 'menstrual':ab,ti OR 'premenstrual syndrome':ab,ti OR 'premenstrual symptoms':ab,ti)

**5 paper**
